# Acute kidney injury comorbidity analysis based on international classification of diseases-10 codes

**DOI:** 10.1186/s12911-024-02435-0

**Published:** 2024-02-03

**Authors:** Menglu Wang, Guangjian Liu, Zhennan Ni, Qianjun Yang, Xiaojun Li, Zhisheng Bi

**Affiliations:** 1 School of Biomedical Engineering, Guangzhou Medical University, Guangzhou, 511436 China; 2 Shenzhen Dymind Biotechnology Co., Ltd, Shenzhen, 518000 China; 3 Guangzhou Women and Children’s Medical Center, Guangzhou Medical University, Guangzhou, 510623 China; 4 Department of Emergency Medicine, the Second Affiliated Hospital, Guangzhou Medical University, Guangzhou, 510260 China

**Keywords:** Acute kidney injury, Comorbidity, Disease network, Organ crosstalk

## Abstract

**Objective:**

Acute kidney injury (AKI) is a clinical syndrome that occurs as a result of a dramatic decline in kidney function caused by a variety of etiological factors. Its main biomarkers, serum creatinine and urine output, are not effective in diagnosing early AKI. For this reason, this study provides insight into this syndrome by exploring the comorbidities of AKI, which may facilitate the early diagnosis of AKI. In addition, organ crosstalk in AKI was systematically explored based on comorbidities to obtain clinically reliable results.

**Methods:**

We collected data from the Medical Information Mart for Intensive Care-IV database on patients aged $\ge$ 18 years in intensive care units (ICU) who were diagnosed with AKI using the criteria proposed by Kidney Disease: Improving Global Outcomes. The Apriori algorithm was used to mine association rules on the diagnoses of 55,486 AKI and non-AKI patients in the ICU. The comorbidities of AKI mined were validated through the Electronic Intensive Care Unit database, the Colombian Open Health Database, and medical literature, after which comorbidity results were visualized using a disease network. Finally, organ diseases were identified and classified from comorbidities to investigate renal crosstalk with other distant organs in AKI.

**Results:**

We found 579 AKI comorbidities, and the main ones were disorders of lipoprotein metabolism, essential hypertension, and disorders of fluid, electrolyte, and acid-base balance. Of the 579 comorbidities, 554 were verifiable and 25 were new and not previously reported. In addition, crosstalk between the kidneys and distant non-renal organs including the liver, heart, brain, lungs, and gut was observed in AKI with the strongest heart-kidney crosstalk, followed by lung-kidney crosstalk.

**Conclusion:**

The comorbidities mined in this study using association rules are scientific and may be used for the early diagnosis of AKI and the construction of AKI predictive models. Furthermore, the organ crosstalk results obtained through comorbidities may provide supporting information for the management of short- and long-term treatment practices for organ dysfunction.

**Supplementary Information:**

The online version contains supplementary material available at 10.1186/s12911-024-02435-0.

## Background

Acute kidney injury (AKI) is a clinical syndrome that affects multiple organs and systems and has a serious impact on patient prognosis [1]. Globally, the in-hospital prevalence of AKI is 10-15%, while in intensive care unit (ICU) patients, it is over 50% [2]. Even if AKI occurs and then goes into remission, it may progress to chronic kidney disease (CKD) and end-stage renal disease (ESRD), requiring long-term dialysis treatment and resulting in longer hospital stays and higher medical costs [3]. In addition, the global burden of AKI-related mortality far exceeds that of mammary cancer, heart failure, and diabetes and has remained high over the past decades [4]. As a result, AKI is considered a worldwide public health problem with a high incidence, high mortality rates, and high health economic costs [1–4].

There is no effective treatment for AKI, but early diagnosis can minimize kidney damage and promote kidney function recovery. However, the main markers of AKI, serum creatinine (Scr) and urine output (Uo), are not effective in diagnosing early AKI [5]. Hence, additional information is urgently needed to aid in the early diagnosis of AKI to allow for early intervention. There have been many studies exploring potential biomarkers for more sensitive and earlier reflection of renal impairment (e.g., cystatin C, interleukin-18, etc.) [6]. However, the focus of this study is to explore the comorbidities of AKI. Analyzing and exploring the relationship between diseases is known as comorbidity analysis [7]. Comorbidity analysis has offered a new approach to disease diagnosis by investigating disease associations [8]. There is growing evidence of a high comorbidity burden of AKI, yet existing research efforts have focused on one disease or several diseases commonly. For example, Dylewska et al. [9] reviewed the medical documentation on patients with AKI and found that hypertension was common in AKI patients. Hapca et al. [10] evaluated the interplay between AKI and CKD in patients with diabetes using observational studies and showed that the development of both diabetes and CKD increased the risk of AKI. Recently, Clercq et al. [11] explored the cardiovascular disease consequences of AKI using systematic evaluation and meta-analysis and showed that patients with AKI were at higher risk of atrial fibrillation, heart failure, acute coronary syndrome and major adverse cardiac events. To our knowledge, no studies have systematically explored the comorbidity patterns of AKI. Studies have shown that association rule mining can be used to discover interesting associations hidden in large datasets [12]. Therefore, this thesis uses a new approach applying association rule mining to comprehensively explore possible comorbidities in AKI through information from the Electronic Health Records (EHR) of AKI patients. Such an exploration may provide a new insight into the comorbidities of AKI and may help to discover previously unidentified relationships among diseases.

The increasing scale of administrative data on patient admissions and discharges currently provides an unprecedented opportunity to assess comorbidity patterns in AKI. Patient diagnostic data contained in administrative datasets, which usually carries a large amount of health information in the form of standardized International Classification of Diseases (ICD) codes, has more available clinical resources, and is more economically viable [13]. ICD codes have great potential for understanding comorbidities. To this end, the primary objective of this study was to explore the comorbidity pattern of AKI using ICD-10 codes. In addition, we constructed a disease network (DN) based on comorbidity results obtained using ICD-10. DNs are a network structure that can be used to reveal potential links between diseases with similar characteristics, providing a theoretical and practical basis for a deeper understanding of disease relationships and promoting personalized medicine [14].

In addition, the results on comorbidity would allow us to gain further insights into organ crosstalk in AKI. Studies have shown that AKI can be triggered by distant non-renal organs, or it can lead to multiple organ failure in distant non-renal organs, resulting in organ crosstalk. Crosstalk between the kidney and distant non-renal organs of the liver, heart, brain, lung, and gut in AKI has been reported, but most evidence for crosstalk between these has been obtained from animal models [15–19], whereas observations in humans have come from a limited number of participants or have focused only on cytokine levels rather than clinically reliable outcomes [20]. Therefore, this study also aimed to systematically investigate crosstalk between the kidney and other distant non-renal organs based on AKI comorbidities results to obtain reliable clinical results of organ crosstalk.

## Methods

### Data introduction

This study involved three databases: the Medical Information Mart for Intensive Care (MIMIC)-IV, the Electronic Intensive Care Unit (eICU), and the Columbia Open Health Data (COHD). MIMIC-IV was used for data analysis, while eICU and COHD served as pivotal resources for validating the results of the analyses.

#### MIMIC-IV

MIMIC-IV records data related to patients admitted to the ICU or emergency department at the Beth Israel Deaconess Medical Center between 2008 and 2019 and has medical health data and records for more than 380,000 inpatients, including approximately 53,000 patients in the ICU. MIMIC-IV provided information on patient vital signs, medication management, laboratory measurements, diagnostic codes, and more [21]. The main data used in this study were laboratory measurements and diagnostic codes for ICU patients over the full period of the study dataset. Patients’ diagnoses in the database were presented as either version 9 or version 10 of the ICD.

#### eICU

eICU is made up of ICU data from many hospitals in the United States, including routine data collected from more than 200,000 patients admitted to ICUs in 2014 and 2015, and has a wealth of high-quality clinical information, including vital signs, laboratory measurements, diagnosis, and more [22]. The primary data utilized were laboratory measurements and diagnoses for all patients throughout the validation dataset period, where diagnoses are presented in the form of ICD codes.

#### COHD

COHD is the Observational Health Data Science and Informatics database at the Columbia University Irving Medical Center. The database contains 36,578 single concepts and 32,788,901 concept pairs from 5,364,781 patients. It provided public access to EHR prevalence and disease co-occurrence frequencies by diseases, drugs, procedures, and demographics [23]. This study used the diagnoses and services provided by the database.

### Data preparation

**Fig. 1:**
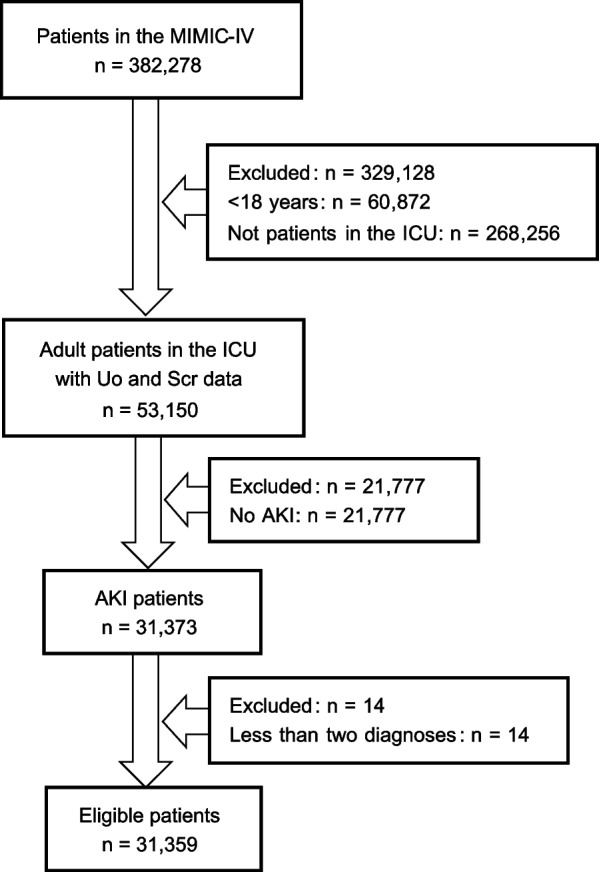
Study flowchart. Flowchart depicts the number of acute kidney injury patients included in the analysis after the exclusion criteria

From laboratory measurements of ICU patients, Scr and Uo were extracted for their ICU stay. AKI was diagnosed and staged according to criteria proposed by Kidney Disease: Improvement Global Outcomes (KDIGO) criteria based on Scr and Uo (Table 1) [24]. In this study, 31,373 AKI patients were identified by definition and their diagnoses were extracted for the period of hospitalization corresponding to when they were identified with AKI. The extracted data were processed using the following methods: 1) As the ICD-10 codes recommended by the World Health Organization have been widely used in the diagnosis of diseases in hospitals, the diagnostic information was represented consistently using ICD-10 codes (ICD-9 codes have been mapped to ICD-10 codes in the diagnosis) [25]. 2) V01-Y98 (external causes of morbidity and mortality) and Z00-Z99 (factors influencing health status and contact with health services) were removed from the patient’s ICD-10 codes, as there may be differences in the criteria for these two categories of codes between countries, which could cause bias in international comparisons [26]. 3) After excluding ICD-10 codes according to Step 2), patients with at least two valid ICD-10 codes were selected, since the study is about associations between diseases where patients with no or only one ICD-10 code do not provide value to the study. As shown in Fig. 1, the final number of patients with AKI included in the analysis after applying the screening and exclusion criteria was 31,359. However, it was not possible to determine whether the comorbidities obtained differed between AKI and non-AKI patients if only AKI patients were analyzed. Therefore, the data analyzed included ICD-10 codes of 55,486 adult patients with AKI and non-AKI. ICD-10 codes can be divided into three levels of affiliation: category, suborder, and detail. The first three digits of the code being the category, such as S52 for fracture of the forearm; The first four digits being the suborder, such as S52.0 for fracture of the upper end of the ulna; Four or more digits being the detail, such as S52.01 for torus fracture of the upper end of the ulna. The first three digits of the ICD-10 code can already basically indicate a specific disease, and more detailed data are not conducive to the presentation of comorbidities, as the number of records with the same disease code is substantially reduced, which is not conducive to uncovering comorbidity patterns of general significance. Therefore, we used the top three digits (categories) of the ICD-10 as the analysis data.

**Table 1:** Measurements used to define and stage AKI

	Scr	Uo
AKI	$\ge$ 0.3 mg/dl ($\ge$ 26.5 µmol/l) increase within 48 hours, or $\ge$ 1.5 times baseline, which is known or presumed to have occurred within the prior 7 days	<0.5 ml/kg/h for 6 hours
Stage 1	1.5-1.9 times baseline, or $\ge$ 0.3 mg/dl (26.5 µmol/l) increase	<0.5 ml/kg/h for 6-12 hours
Stage 2	2.0-2.9 times baseline	<0.5 ml/kg/h for $\ge$ 12 hours
Stage 3	$\ge$ 3.0 times baseline, or increase in creatinine to $\ge$ 4.0 mg/dl (353.6 µmol/l)	<0.3 ml/kg/h for $\ge$ 24 hours, or anuria for $\ge$ 12 hours

### Statistical analysis

Demographic characteristics (sex, age, and ethnicity) of AKI and non-AKI were compared using chi-square tests. To facilitate the study, patients divided into three groups according to age, i.e., youth (18-35 years of age), middle age (36-59 years of age), and old age ($\ge$ 60 years). In addition, the diagnostic information of AKI patients was statistically analyzed and visualized in this study. Significance of differences between subgroups was compared using t-tests (continuous variables), ANOVA (continuous variables) and chi-square tests (discrete variables). A value of *p* < 0.05 was considered to be statistically significant.

### Disease rules mining

Association rule mining is used to discover interesting itemset associations hidden in large datasets that are usually represented in the form of association rules or frequent itemsets. In general, if there is an association between two or more transactions, the occurrence of one transaction can predict the occurrence of the other transactions associated with it [12]. Therefore, the results of association rule mining can often be used to build predictive models. The aim of this study is to mine association rules for diseases by frequent 2-itemset. Apriori algorithm can obtain frequent binomial sets based on frequent 1-itemset, while FP-growth algorithm must first aggregate all frequent sets before filtering the frequent 2-itemset. In other words, Apriori algorithm is more efficient at obtaining frequent 2-itemset. Therefore, we used Apriori algorithm for comorbidity mining.

In this study, three indicators support (sup), kulczynski (kulc), and lift (sup:8; kulc:0; lift:1) were used to measure the degree of association of comorbidity pairs. The formulas for the three indicators are as follows [27].

1 $$\begin{aligned} sup\left( A\rightarrow B \right) \,\,=\,\,P\left( A\cup B \right) \end{aligned}$$

2 $$\begin{aligned} kulc\left( A,B \right) =\,\,\frac{P\left( A|B \right) \,\,+\,\,P\left( B|A \right) }{2} \end{aligned}$$

3 $$\begin{aligned} lift\left( A\rightarrow B \right) =\frac{P\left( B|A \right) }{P(B)}=\frac{P\left( A\cup B \right) }{P\left( A \right) *P(B)} \end{aligned}$$

$sup\left( A\rightarrow B \right)$ represents the proportion of records with concurrent disease A and B to the total diagnostic records; that is, the probability that A and B occur together.

$kulc\left( A,\,\,B \right)$ represents the mean value of the confidence with diseases A and B as conditions and is used to measure the reliability of A and B as comorbidities of each other.

$lift\left( A\rightarrow B \right)$ represents the ratio of the probability of occurrence of disease B in a record containing disease A to the probability of occurrence of disease B itself and is used to measure the relevance of A and B.

### Comorbidities verification

To demonstrate the scientific validity of mined AKI comorbidities, the results were validated primarily using the eICU and the COHD. In addition, medical literature reviewed by Google Scholar and Pubmed was used as an aid to validate in this study.

First validated using a large publicly available eICU database. The same methods were used here to process and analyze the eICU data. Firstly, 52,869 adult AKI patients were recruited from the eICU according to the KDIGO criteria (Table 1), and diagnostic information was obtained for a total of 95,428 patients including adult non-AKI patients. Secondly, the data was processed and then mined for association rules using Apriori algorithms. Finally, mining results from the eICU (Supplementary Table 1) were compared with those from MIMIC-IV. If comorbidity pairs mined in MIMIC-IV are also present in the eICU upon enquiry, comorbidity pairs can be proven by the eICU. For example, N17 (acute kidney injury) and I10 (essential hypertension) were an interesting comorbidity pair from the MIMIC-IV database, which was also one of the results of eICU mining. Then the comorbidity pair (N17, I10) could be confirmed by the eICU.

The second proving method used the publicly accessible COHD. It provides direct access to the comorbidity pair analysis results (Chi-square, relative frequency, and observed-expected frequency ratio) [28], thus enabling validation of the results of this study. However, the disease codes used for COHD analysis are the Observational Medical Outcomes Partnership Common Data Model concept ID, which are different from the ICD-10 codes used in this study. Therefore, the mapping tool [29] provided by COHD was used to map ICD-10 to OMOP concept ID (Supplementary Table 2). For validation of the comorbidity results, chi-square tests were used. If a comorbidity pair was present in COHD and was significant (*p* < 0.05), the comorbidity pair was considered true. However, not all ICD-10 codes exactly matched the OMOP concept ID. Therefore, match validation was divided into three steps. For purposes of argument, the first three and four digits of ICD-10 were referred to as ID-3 and ID-4, respectively, and five or more digits were referred to as ID-5. First, matching was performed using comorbidity ID-3. COHD was used for direct validation of ID-3 that could be matched to OMOP Concept ID. Second, the mismatch ID-3 for each patient in the comorbidity analysis dataset was replaced with its corresponding ID-4 and association rule mining was performed again using the replaced dataset. For example, S09 could not be matched and a patient’s original diagnostic code had S09.11 and S09.31, S09.1 and S09.3 were used to replace S09 for that patient. The comorbidity ID-4 obtained from the analysis was then matched and proven. If any ID-4 could be proven as comorbidity by COHD, its corresponding ID-3 was also proven as comorbidity. For example, as long as at least one of S09.1 and S09.3 can be demonstrated at COHD to be a comorbidity of AKI, the comorbidity pair (N17, S09) was considered to be demonstrable by COHD. Moreover, for ID-4, which also did not match the OMOP concept ID, ID-3 in the dataset was replaced with the corresponding ID-5. The association rule analysis was performed again, and the analyzed comorbidity, ID-5, was matched and proved.

Finally, medical literature (Supplementary Table 3) was reviewed to identify comorbidity pairs that could not be confirmed by either of the above methods. AKI was previously known as Acute Renal Failure (ARF), so using either AKI or ARF as well as unproven comorbidity as keywords in Google Scholar and PubMed was used to review the evidence.

### Disease networks

**Fig. 2:**
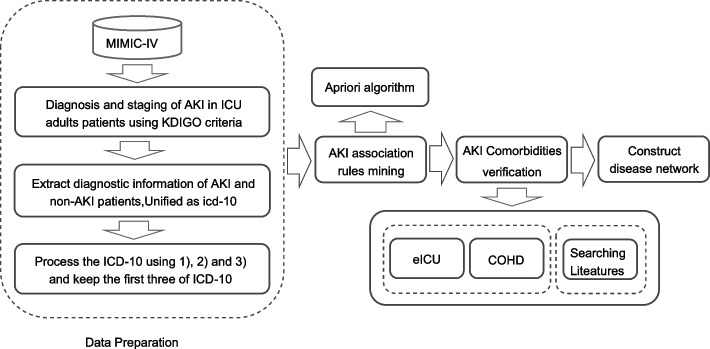
Procedures and steps used in comorbidity studies. There are four main steps: Data preparation, acute kidney injury association rule mining, acute kidney injury comorbidities verification, and construction of disease networks

Based on the comorbidity results mined in this study, a DN was constructed. A node in a DN represents a disease (ICD-10), and node colors are classified based on the ICD-10 chapters, with the size indicating the importance of the disease. The width of the node edges indicates kulc, and the larger the width the larger the kulc. The color of the node edges from dark to light indicates lift from large to small. Figure 2 summarizes the technical route of the method used in AKI comorbidity studies.

### Organ crosstalk in AKI

Based on the results obtained for AKI comorbidities, classification studies were conducted. First, we classified all AKI comorbidities by ICD-10 chapters to explore which systemic diseases in comorbidities had a strong association with AKI. Diseases associated with distant non-kidney organs (Supplementary Table 4) were then screened and classified, and organ crosstalk in AKI was systematically explored to obtain clinically reliable results. In this study, the following formula was devised to calculate the importance of the relationship between a certain classification and AKI:

4 $$\begin{aligned} score_u\,\,=\,\, {\sum \limits _{j\epsilon u}{sup\left( AKI,\,\,j \right) }} \,\,\times \,\,kulc\left( AKI,\,\,j \right) \end{aligned}$$

*u* represents a classification, such as heart disease; *j* represents all sub-items in *u*. *sup* and *kulc* represent the frequency and credibility of comorbidity pairs, respectively, with higher values indicating greater comorbidity importance. The number of comorbidities included in a classification, in addition to *sup* and *kulc*, is an important indicator of the extent to which a classification affects AKI. According to equation (4), as *sup*, *kulc*, and the number of comorbidities in the classification increase *score* is greater. Therefore, it is reasonable to use it to calculate the degree of association between disease categories and AKI.

## Results

### Demographic characteristics

As shown in Table 2, higher incidence of AKI in males (60.59 %) compared to females (*p* < 0.001). Stage 2 of AKI was more prevalent in females, and the remaining two stages followed the overall trend (*p* < 0.001). For the age distribution of patients, the highest incidence of AKI was observed in the elderly (72.21%), and the incidence of AKI increased with age stage (*p* < 0.001). Similarly, the prevalence of each stage of AKI is highest among older adults. In addition, a comparison of the ethnicity of the patients showed that, excluding other races and unknown races, the incidence was highest in Caucasians and lowest in Asians (*p* < 0.001). Stage 1 of AKI continues to have a high prevalence among Caucasians, while stages 2 and 3 have a higher prevalence among Native Americans (*p* < 0.001).

**Table 2:** Basic demographic characteristics of AKI patients included in the study

Characteristic	AKI, N^a^ (%)	AKI-I, N (%)	AKI-II, N (%)	AKI-III, N (%)
**Sex**				
Male	17965 (60.29%)	12770 (42.86%)	4346 (14.59%)	849 (2.85%)
Female	13394 (57.35%)	9375 (40.14%)	3498 (14.98%)	521 (2.23%)
*P*-value	< 0.001	< 0.001		
**Age**				
18-35	1368 (33.91%)	1013 (25.11%)	281 (6.97%)	74 (1.83%)
36-59	7347 (51.70%)	5119 (36.02%)	1867 (13.14%)	361 (2.54%)
$\ge$ 60	22644 (64.88%)	16013 (45.88%)	5696 (16.32%)	935 (3.68%)
*P*-value	< 0.001	< 0.001		
**Ethnicity**				
Black/African American	2837 (56.79%)	1808 (36.19%)	747 (14.95%)	282 (5.64%)
White	21840 (59.91%)	15548 (42.65%)	5499 (15.09%)	793 (2.18%)
Asian	735 (45.91%)	538 (33.60%)	151 (9.43%)	46 (2.87%)
Hispanic/Latino	983 (51.68%)	676 (35.54%)	240 (12.62%)	67 (3.52%)
American Indian/Alaska Native	57 (58.76%)	35 (36.08%)	16 (16.49%)	6 (6.19%)
Other	1116 (53.84%)	797 (38.45%)	285 (13.75%)	34 (1.64%)
Unknown	3791 (62.88%)	2743 (45.50%)	906 (15.03%)	142 (2.36%)
*P*-value	< 0.001	< 0.001		

^a^ *N* = number

### Diseases in adults with AKI

**Fig. 3:**
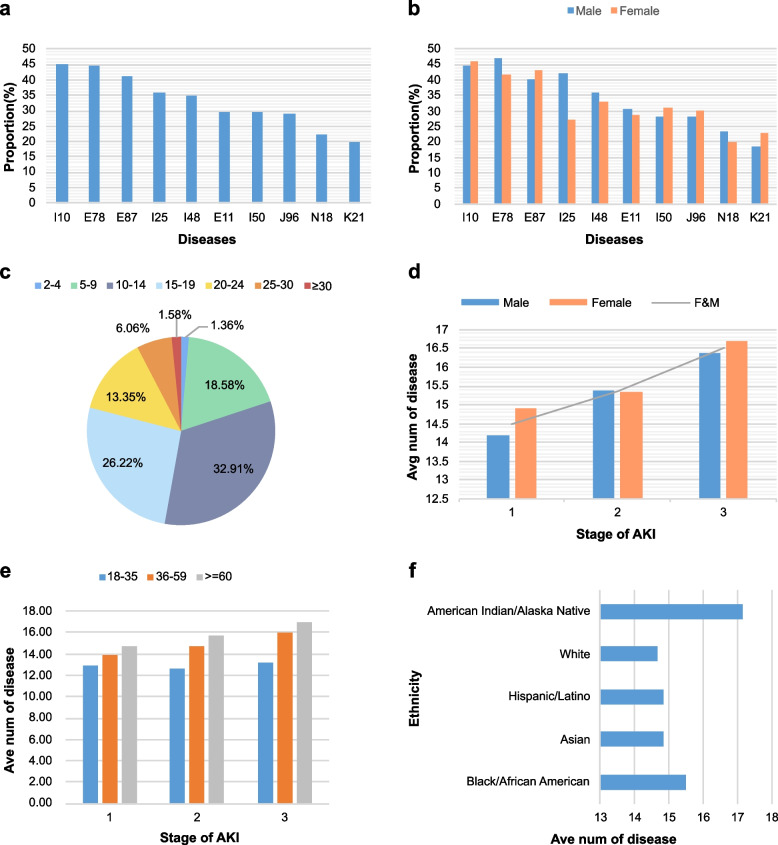
Basic clinical characteristics of 31,359 patients with acute kidney injury are included in this study. **a** Top 10 common diseases in acute kidney injury; **b** Sex distribution of ten common diseases; **c** Number distribution of diseases among acute kidney injury patients; **d** Sex distribution of the mean number of diseases based on different acute kidney injury stages. **e** Age distribution of the mean number of diseases based on different acute kidney injury stages. **f** Ethnic distribution of mean number of diseases in patients with acute kidney injury. * I10: Essential hypertension; E78: Disorders of lipoprotein metabolism; E87: disorders of fluid, electrolyte, and acid-base balance; I25: Chronic ischemic heart disease; I48: Atrial fibrillation and flutter; E11: Type 2 diabetes mellitus; I50: Heart failure; J96: Respiratory failure; N18: Chronic kidney disease; K21: Gastro-esophageal reflux disease

In total 1,291 diseases other than AKI were identified from diagnostic information of 31,359 patients with AKI, and statistical analysis of this diagnostic data follows. Figure 3a illustrates the ten most frequent diseases, the most frequent of which are essential hypertension (I10); disorders of lipoprotein metabolism and other lipidemias (E78); disorders of fluid, electrolyte, and acid-base balance (E87); and chronic ischemic heart disease (I25) (*p* < 0.001). Of these ten diseases, E78 (*p* < 0.001), I25 (*p* < 0.001), I48 (*p* < 0.001), E11 (*p* < 0.001), N18 (*p* < 0.001) were highly prevalent in males with AKI, especially I25, while I10 (*p* < 0.005), E87 (*p* < 0.001), I50 (*p* < 0.001), J96 (*p* < 0.001), and K21 (*p* < 0.001) were more prevalent in females (Fig. 3b). The number of diseases in patients with AKI was also compared. The highest proportion of patients had 10-14 diseases simultaneously (32.91%), followed by those with 15-19 diseases (26.22%) (*p* < 0.001). Notably, only 1.36% of patients had 2-4 diseases, further indicating that AKI is a difficult clinical syndrome, often accompanied by a large number of complications. The number of diseases tended to increase as AKI became more severe (1 vs 2, *p* < 0.001; 1 vs 3, *p* < 0.001; 2 vs 3, *p* < 0.001). The number of diseases was higher in females than in males in stage 1 AKI (*p* < 0.001), and the difference between genders in the other two stages was not significant (*p*>0.05). Age was also positively correlated with the number of diseases, with older AKI patients having the highest number of combined other diseases (youth vs middle, *p* < 0.001; youth vs old, *p* < 0.001; middle vs old, *p* < 0.001). And the different stages of AKI were consistent with the overall trend (Fig. 3e). In addition, Native Americans had more combined diseases than AKI patients of any other race (*p* < 0.05), except Black (*p*>0.05), who had more than Hispanics, Latinos, and Asians (*p* < 0.05) (Fig. 3f).

### Comorbidity mining results and disease network

A total of 579 AKI comorbidities were obtained using the Apriori algorithm, of which approximately 37% (213/579) of the associations were provable in the eICU. COHD allowed verification of 88% (511/579) of comorbidities, 182 of which were also included in the eICU. Using both methods, more than 94% (542/579) of comorbidities were demonstrable. Among the remaining 39 comorbidities, 14 could be proven by reviewing the literature, but 25 could not (Table 3) (Supplementary Table 5). This indicates that the comorbidities we mined are scientific. The main categories of diseases among numerous comorbidities were circulatory system diseases, endocrine system diseases, and respiratory system diseases, as shown in Fig. 5. In addition, 284 high incidence comorbidities were selected from which to construct a DN for simplicity and clarity of visualization (Fig. 6). The largest nodes in this network were disorders of lipoprotein metabolism and other lipidemias (E78); essential hypertension (I10); disorders of fluid, electrolyte, and acid-base balance (E87); and atrial fibrillation and flutter (I48). This finding is similar to that in the common diseases in AKI patients, as shown in Fig. 3, except for a slight change in ranking due to kulc. In addition, anuria and oliguria (R34), acute respiratory distress syndrome (J80), and intraoperative and postprocedural complications and disorders of the respiratory system (J95) had the darkest margins, indicating that they were more strongly associated with AKI.

**Table 3:** Consistency of the results of this study with those in eICU, COHD and medical literature

Confirmed by eICU	Confirmed by COHD			Total
	Yes	No		
Yes	182	31		213
No	327	As reported in the literature (39)		366
		Yes	No	
		14	25	
Total	509	70		579

**Fig. 4:**
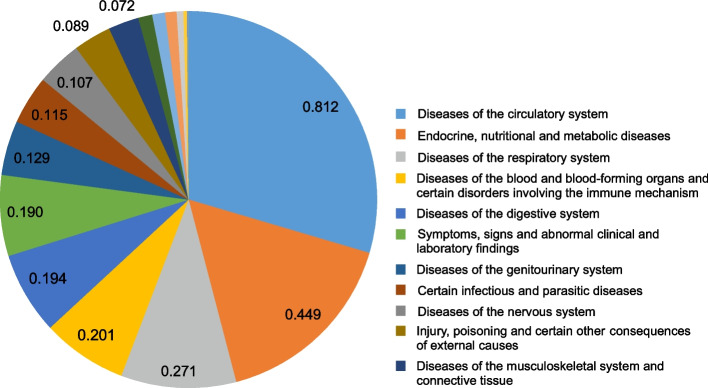
Systems to acute kidney injury correlation. Classifications based on ICD-10 chapters

**Fig. 5:**
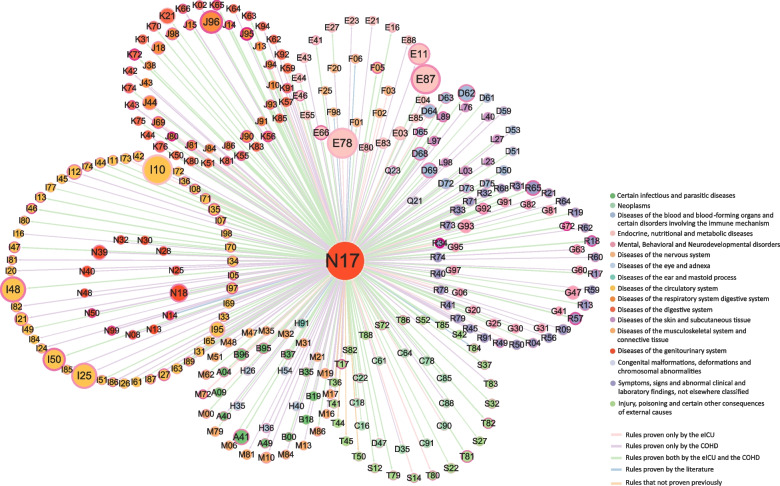
Disease network. Nodes represent diseases, with different colors representing the disease classification based on ICD-10 sections and sizes become larger as sup * kulc increases. The width of the node border indicates the kulc of the disease and acute kidney injury, while the color ranges from dark to light to indicate a greater to lesser correlation between the two diseases. The color of the link indicates the different results of comorbidities verification

### Risk factors for severe AKI

Which diseases were risk factors for stage 3 AKI was explored by comparing comorbidities at different stages of AKI. Studies have shown that severe AKI was associated with increased incidence of CKD, ESRD, and increased mortality [30–32]. By understanding the risk factors for severe AKI (stage 3), it may be possible to predict it in advance and intervene in time to treat it, ultimately reducing the healthcare burden. AKI was classified as stage 1 (22,145), stage 2 (7,844), or stage 3 (1,370) according to the KDIGO criteria (Table 1). Comorbidities were analyzed for different stages of AKI and selected the factors that uniquely distinguished severe AKI as risk factors. For example, E10 has been analyzed as a comorbidity of stage 3 AKI and not stages 1 and 2, so the presence of E10 may be associated with the development of severe AKI. As shown in Fig. 4, type 1 diabetes mellitus (E10) was the largest node with a higher frequency of co-occurrence with severe AKI. Based on the nodal edge color depth, the contracted kidney (N26) was the darkest with the strongest correlation for severe AKI.

**Fig. 6:**
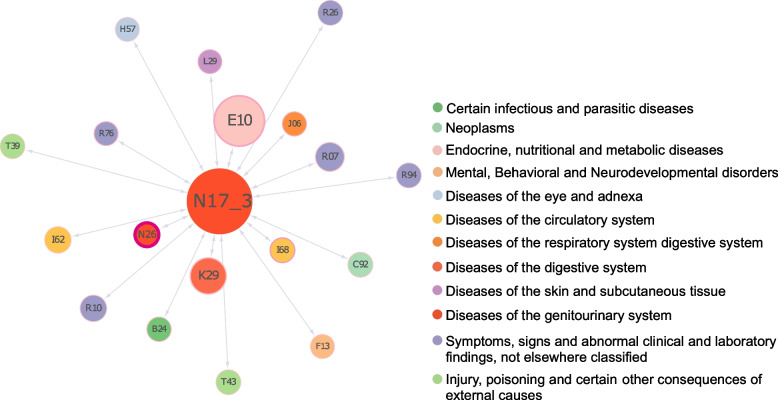
Disease networks with severe acute kidney injury risk factors. Nodes indicate diseases, their size indicates importance, and their color indicates the type of disease. The width of the node border indicates kulc and the color indicates the correlation

### Distant organ effects in AKI

**Fig. 7:**
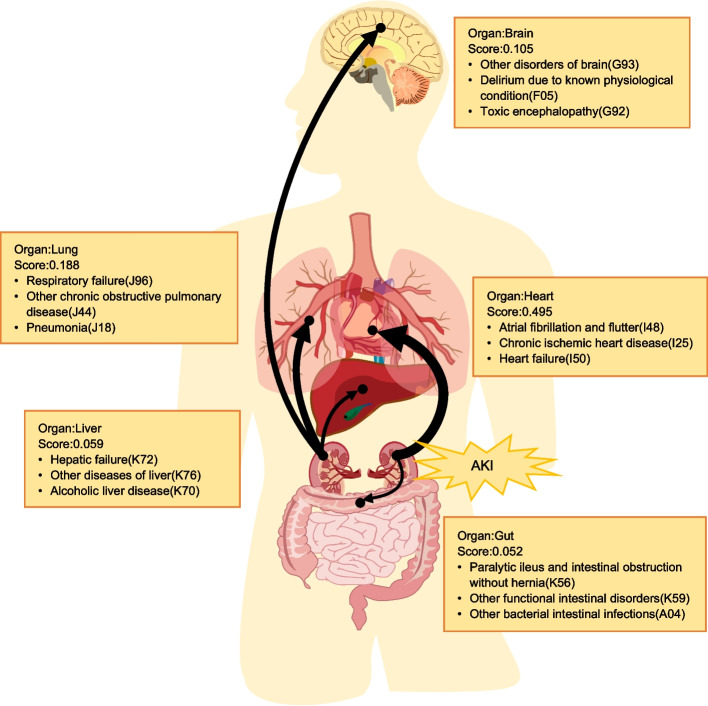
Organ interactions in acute kidney injury. The width of the link indicates the degree of crosstalk (score), while the text displays the score for each crosstalk and the top three comorbidities in the crosstalk

Comorbidity-based study has led to further clinical validation of the presence of crosstalk between the kidneys and distal non-renal organs in AKI. Of all the crosstalk, the heart-kidney crosstalk was the strongest and much larger than the lung-kidney crosstalk, which came in second (Fig. 7). Common heart diseases in crosstalk were atrial fibrillation and flutter (I48), chronic ischaemic heart disease (I25), and heart failure (I50), while lung diseases were respiratory failure, chronic obstructive pulmonary disease and pneumonia.

## Discussion

In this study, a comprehensive study of comorbidities in AKI was conducted using the Aprior algorithm. The study identified 579(554/579) comorbidities, 96% of which were validated by COHD(511/579), eICU(231/579), and medical literature(24/37). This shows that mining comorbidities using the Aprior algorithm is feasible and the parameters used are reasonable [33], thus making the mining results scientifically valid. Comorbidities mainly involved circulatory, endocrine and respiratory diseases, which meant that AKI had clear correlations with them. Although this study did not delve into the underlying mechanisms and interrelationships between AKI and these three systems, it may provide direction for future research. In addition, this study constructed a DN based on comorbidities, which were used to gain a deeper understanding of disease associations. The largest nodes in the DN include disorders of lipoprotein metabolism and other lipidemias(E78), essential hypertension(I10), disorders of fluid, electrolyte, and acid-base balance(E87), and atrial fibrillation and flutter(I48). These comorbidities have been mentioned in previous studies [34–38]. Such as Druml et al. [34] claimed that the occurrence of AKI disorders lipoprotein metabolism; Dylewska et al. [35] assessed the prevalence of hypertension in patients with AKI and showed that hypertension is common in AKI. This further demonstrates the reliability of the results.

Additionally, 25 new AKI comorbidities were identified in this study. Studies have shown that the inclusion of new comorbidities increases the overall understanding of AKI and contributes to improved prediction and decision-making in AKI, leading to the designation of more effective treatment strategies for better management of the patient’s health status [39, 40]. Although they have not been directly confirmed, the information they hide could be useful. Notably, four of the 25 new comorbidities were associated with drug intoxication (T36, T44, T45, and T50). The kidney is particularly susceptible to the effects of drugs because it is the main excretory organ of the body. Some drugs can induce AKI through various pathophysiological pathways, leading to acute nephrotoxic kidney injury [41]. Three of the new comorbidities were malignant neoplasms (C02, C44, and C54). Consistently, studies have shown that the incidence of AKI in patients with malignancies is as high as 12%, and there are many causes of AKI in malignancies, such as cancer, cancer-related metabolic disorders, anti-cancer treatment, and other complications [42]. In addition, some of the new comorbidities, although not directly demonstrable in literature, could be reviewed to find their association with AKI, such as other zoonotic bacterial diseases (A28) and nonscarring hair loss (L65). The former (A28) contains pasteurellosis (A28.0) and cat-scratch disease (A28.1). There is a greater association between these two zoonotic diseases and sepsis [43], while sepsis is closely related to the development of AKI [44]. The latter (L65) can be caused by metabolic disorders [45], whereas AKI can lead to the development of metabolic diseases [46]. Collectively, these results suggest new comorbidities in AKI, and they may be more or less associated with AKI.

This study also explored crosstalk between the kidney and distant non-renal organs (including liver, heart, brain, lungs, and gut) in AKI based on comorbidities. Although previously reported in the literature, it was mostly based on evidence obtained from animal models [15–19]. This study obtained evidence based on clinical data leading to clinically reliable results and compared the intensity of crosstalk. The results showed that heart-kidney crosstalk was the strongest, followed by lung-kidney crosstalk, and the intensity of heart-kidney crosstalk was much higher than lung-kidney crosstalk. Therefore, the monitoring of both heart and kidney organ diseases should be strengthened in clinical practice to help early detection and management of heart and kidney problems in patients, and to improve therapeutic efficacy and patients’ quality of life.

Despite our best efforts, there are limitations to this study. First, ICD codes are mainly concerned with the diagnosis and classification of diseases and do not involve deeper biological information such as disease pathogenesis and molecular changes. Therefore, in future work, molecular data, such as genomics and proteomics, can be combined with ICD data to better explain disease associations and understand the underlying biological mechanisms, thus revealing the reasons behind the onset and progression of comorbidities [47]. The incorporation of molecular data not only provides a deeper understanding but also helps identify potential biomarkers, providing more targeted information for personalized medical care and treatment. Second, disease associations obtained using association rule mining can only indicate coexistence between diseases, not causation. However, in future practice, disease associations can be studied in a more comprehensive and in-depth manner, taking into account the sequence of diseases and expertise in the medical field. This will enable a more accurate determination of causation and an understanding of which disease may lead to another. Medical expertise can provide an in-depth understanding of the physiological and pathophysiological characteristics of diseases, can reveal possible causal links between events, and can help establish more reliable inferences about relationships. While temporal relationships between disease occurrences can provide additional clues to help understand whether a causal relationship exists. In conclusion, this approach provides a more in-depth and interpretable direction for future research. In conclusion, this integrated approach to research provides a deeper and more interpretable direction for future research.

## Conclusion

In this study, the comorbidity of AKI was investigated by association rule mining. The validation results of comorbidities not only indicate that comorbidities have scientific validity, which may provide references for clinical diagnosis of AKI and the construction of AKI risk prediction models, but also indicate that mining comorbidities using association rules is feasible. In addition, organ crosstalk in AKI was systematically investigated based on comorbidity results, and clinically reliable results were obtained.

### Supplementary information

**Additional file 1.**

## Data Availability

A The dataset used in this study can be accessed in the mimic-iv repository, https://physionet.org/content/mimiciv/0.4/.

## Abbreviations

AKI: Acute Kidney Injury
CKD: Chronic Kidney Disease
ESRD: End Stage Renal Disease
COHD: Colombian Open Health Database
DNs: Disease Networks
eICU: Electronic Intensive Care Unit
EHR: Electronic Health Records
ICD: International Classification of Diseases
KDIGO: Kidney Disease Improvement Global Outcomes
MIMIC: Medical Information Mart for Intensive Care
Uo: Urine Output
Scr: Serum Creatinine
